# Loss of function of *Ywhah* in mice induces deafness and cochlear outer hair cells' degeneration

**DOI:** 10.1038/cddiscovery.2016.17

**Published:** 2016-03-07

**Authors:** L Buret, G Rebillard, E Brun, C Angebault, M Pequignot, M Lenoir, M Do-cruzeiro, E Tournier, K Cornille, A Saleur, N Gueguen, P Reynier, P Amati-Bonneau, A Barakat, C Blanchet, P Chinnery, P Yu-Wai-Man, J Kaplan, A-F Roux, G Van Camp, B Wissinger, O Boespflug-Tanguy, F Giraudet, J-L Puel, G Lenaers, C Hamel, B Delprat, C Delettre

**Affiliations:** 1 INSERM U1051, Institute of Neurosciences of Montpellier, Montpellier, France; 2 Department of Biology and Health Sciences, University of Montpellier, Montpellier, France; 3 Homologuous Recombination, Cochin Institute, University Paris Descartes, Paris, France; 4 Department of Ophthalmology, Angers University Hospital, Angers, France; 5 Laboratory of Human Genetic and Molecular Biology, Pasteur Institute, Casablanca, Morocco; 6 CHRU Montpellier, Centre of Reference for Genetic Sensory Diseases, CHU, Gui de Chauliac Hospital, Montpellier, France; 7 Mitochondrial Research Group, Institute of Ageing and Health, The Medical School, Newcastle University, Newcastle, UK; 8 Laboratory of Genetics in Ophthalmology, Inserm UMR1163, Institut Imagine, Université Paris Descartes Sorbonne Paris Cité, Hôpital Necker, Paris, France; 9 Laboratory of Molecular Genetic, CHU Arnaud de Villeneuve, Montpellier, France; 10 Center of Medical Genetics, Department of Biomedical Sciences, University of Antwerp, Antwerp, Belgium; 11 Molecular Genetics Laboratory, Institute for Ophthalmic Research, Tuebingen, Germany; 12 Neurobiology and Metabolic Diseases, Hospital Robert Debré, Paris, France; 13 Laboratory of Neurosensorial Biophysic, UMR INSERM 1107, Faculty of Medicine – Auvergne University, Clermont Ferrand, France

## Abstract

In vertebrates, 14-3-3 proteins form a family of seven highly conserved isoforms with chaperone activity, which bind phosphorylated substrates mostly involved in regulatory and checkpoint pathways. 14-3-3 proteins are the most abundant protein in the brain and are abundantly found in the cerebrospinal fluid in neurodegenerative diseases, suggesting a critical role in neuron physiology and death. Here we show that 14-3-3eta-deficient mice displayed auditory impairment accompanied by cochlear hair cells' degeneration. We show that 14-3-3eta is highly expressed in the outer and inner hair cells, spiral ganglion neurons of cochlea and retinal ganglion cells. Screening of *YWHAH*, the gene encoding the 14-3-3eta isoform, in non-syndromic and syndromic deafness, revealed seven non-synonymous variants never reported before. Among them, two were predicted to be damaging in families with syndromic deafness. *In vitro*, variants of *YWHAH* induce mild mitochondrial fragmentation and severe susceptibility to apoptosis, in agreement with a reduced capacity of mutated 14-3-3eta to bind the pro-apoptotic Bad protein. This study demonstrates that *YWHAH* variants can have a substantial effect on 14-3-3eta function and that 14-3-3eta could be a critical factor in the survival of outer hair cells.

## Introduction

The 14-3-3 proteins were initially described as tyrosine 3-monooxygenase/tryptophan 5-monooxygenase activation proteins (YWHAx) required for the synthesis of neurotransmitters (dopamine, (nor-) adrenaline, serotonin) from catecholamine.^1^ Subsequently, studies described the ability of 14-3-3 proteins to form homo- or hetero-dimeric complexes and numerous reports of new binding partners confirmed their chaperone activity.^2,3^ This highly conserved family of small 28–33 kDa acidic dimeric proteins consists of seven distinct subunit isoforms (*β*, *ε*, *γ*, *η*, *τ*, *σ* and *ζ*) encoded by seven genes named *YWHAx*, (x being either B, E, G, H, Q, S or Z).^4^ These proteins mostly bind serine/threonine phosphorylated ligands altering their sub-cellular localization, stability, phosphorylation state, activity or molecular interactions with other targets, thus controlling cell cycle and many signal transduction pathways.^2,3^ A primary function of 14-3-3 proteins is the inhibition of apoptosis by retaining pro-apoptotic factors such as Bad or Bax in the cytoplasm.^5,6,7^ 14-3-3 proteins were originally discovered as abundant molecules in the brain,^8^ and follow-up studies confirmed that the highest tissue concentration of 14-3-3 proteins is in the brain,^9^ comprising about 1% of total proteins of the brain. In the peripheral nervous system, proteomics experiments showed that 14-3-3 proteins were expressed in the cochlea,^10,11,12^ and among the seven 14-3-3 isoforms, 14-3-3eta encoded by the *YWHAH* gene has been found highly expressed in retinal ganglion cells (RGC).^13^ The crucial involvement of 14-3-3 proteins in neuronal physiology led to investigate them in the pathophysiology of neurological diseases as well as considering the *YWHAx* genes as candidate genes in neurodegenerative conditions.^14,15,16,17,18^ Subsequently, 14-3-3 proteins' involvement was confirmed in a number of neurological disorders, including Parkinson disease, amyotrophic lateral sclerosis, Alzheimer disease, epilepsy and Creutzfeldt–Jakob disease,^19^ but whether it is involved in sensory organ dysfunction or degeneration remains unknown. Here we have investigated the function of 14-3-3eta in both auditory and visual systems, and we report that the loss of 14-3-3eta protein is associated with cochlear hair cells' degeneration.

## Results

### *Ywhah* loss of function induces deafness in mice

To investigate the role of 14-3-3eta in optic and auditory systems, we obtained an embryonic stem cell line harboring a genetrap (GT) cassette in intron 1 of the *Ywhah* mouse gene and generated the corresponding transgenic mouse. Processing of the mRNA transcribed from this locus resulted in the fusion of the *β*-galactosidase/neomycin cassette to exon 1, generating a 14-3-3eta truncated protein (Supplementary Figure S1a). *Ywhah* expression level (Supplementary Figures S1b and c) and 14-3-3eta protein abundance (Supplementary Figure S1d) were decreased by 50% in 14-3-3eta^GT/WT^ mice and were absent in 14-3-3eta^GT/GT^ mice. Mice were morphologically normal and had a normal lifespan but 14-3-3eta^GT/GT^ males were sterile.

We first assessed the visual function of those mice as 14-3-3eta is highly expressed in RGC.^13^ Electroretinograms were recorded in animals from 2 to 16 months of age. Both A- and B-wave amplitudes tend to decrease from 12 months of age in 14-3-3eta^GT/GT^ mice in comparison to 14-3-3eta^WT/WT^ and 14-3-3eta^GT/WT^ animals, but this did not reach significance (Supplementary Figure S2a). There was no change in latencies. Measurements of the visual evoked potentials that reflect the integrity of the visual pathway including RGC did not reveal significant difference between 14-3-3eta^GT/WT^, 14-3-3eta^GT/GT^ mice and 14-3-3eta^WT/WT^ littermate (Supplementary Figure S2b). Accordingly, counting of RGC by Brn3a labeling did not evidence difference between 14-3-3eta^GT/GT^ and 14-3-3eta^WT/WT^ mice (Supplementary Figure S2c).

**Figure 1:**
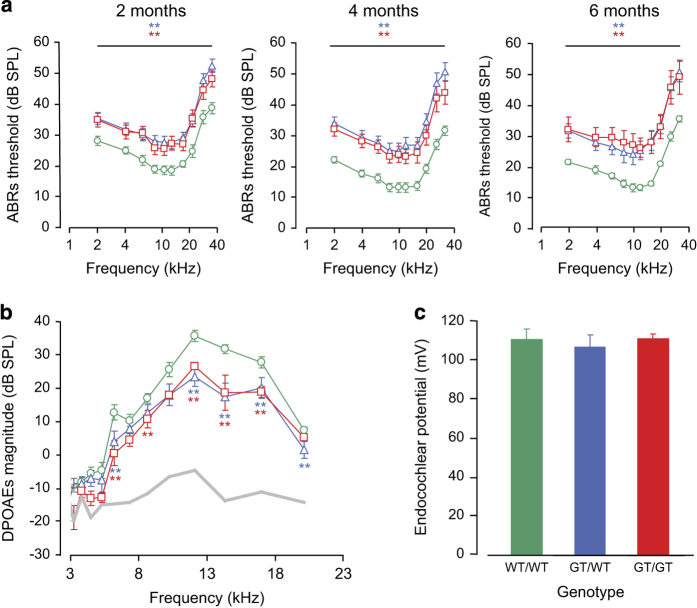
Invalidation of Ywhah induces a mild deafness but does not affect the functioning of the stria vascularis. (**a**) Audiograms established in 14-3-3eta^WT/WT^ (*n*=18), 14-3-3eta^GT/WT^ (*n*=16) and 14-3-3eta^GT/GT^ (*n*=20) by measuring the brainstem evoked potential thresholds at 10 frequencies ranging from 2 to 32 kHz. Data show a mild (10–20 decibels (dB)) but very significant (*P*<0.01 for all tested frequencies) threshold elevation in 14-3-3eta^GT/WT^ (blue triangles) and 14-3-3eta^GT/GT^ (red squares) mice as compared with 14-3-3eta^WT/WT^ (green circles) at 2, 4 and 6 months. (**b**) This panel displays cubic DPOAE (2*f*_1_−*f*_2_) audiograms represented as a function of *f*_2_. The magnitude of the distortion product is slightly reduced in 14-3-3eta^GT/WT^ (blue triangles; *n*=14) and 14-3-3eta^GT/GT^ (red squares; *n*= 6) as compared with 14-3-3eta^WT/WT^ mice (green circles; *n*=14). This reduction is significant between 6 and 20 kHz. The gray line represents the noise floor. (**c**) Endocochlear potentials were recorded in 14-3-3eta^WT/WT^ (green bar; *n*=5), 14-3-3eta^GT/WT^ (blue bar; *n*=5) and 14-3-3eta^GT/GT^ mice (red bar; *n*=5). No significant difference was seen between the three genotypes. Data are mean±S.E.M.

We then assessed the auditory acuity by recording the auditory brainstem responses (ABRs) in animals from 2 to 16 months of age to determine the threshold at 10 frequencies ranging from 2 to 32 kHz corresponding to the apical half of the mouse cochlea (Figure 1). All mutant animals (14-3-3eta^GT/WT^ (*n*=16) and 14-3-3eta^GT/GT^ (*n*=20)) had a significant (0.006<*P*<0.02) and stable 15–20 dB threshold shift compared with 14-3-3eta^WT/WT^ animals (*n*=18) (Figure 1a). Distortion product oto-acoustic emissions (DPOAEs), which reflect the non-linear amplification of the outer hair cells (OHCs) on the basilar membrane motion, were also decreased in 14-3-3eta^GT/WT^ (*n*=12) and 14-3-3eta^GT/GT^ (*n*=6) compared with 14-3-3eta^WT/WT^ (*n*=14), with significant values between 6 and 20 kHz (0.001<*P*<0.03), again independently of animal age (Figure 1b). Conversely, no effect of the *Ywhah* mutant (*P*>0.54) was observed on the endocochlear potentials of 14-3-3eta^GT/WT^ (106.8±6.45 mV, *n*=5), 14-3-3eta^GT/GT^ (111.4±1.99 mV, *n*=5) and the 14-3-3eta^WT/WT^ (110.8±5.5 mV, *n*=5) (Figure 1c).

### *Ywhah* is critical for OHCs' survival

**Figure 2:**
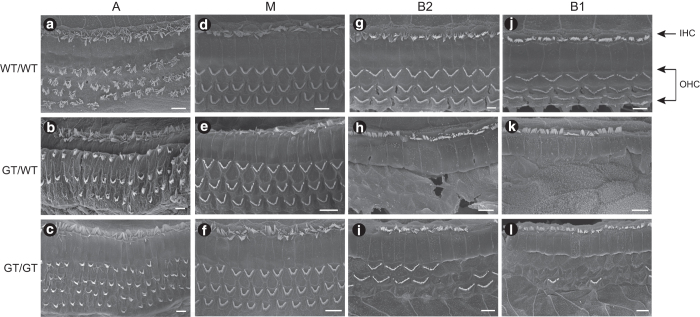
Degeneration of cochlear cells in 14-3-3eta mutant mice. Scanning electron microscopy of the organ of Corti at the apical turn (A; **a**–**c**), the middle turn (M; **d**–**f**) , the upper basal turn (B2; **g**–**i**) and the lower basal turn (B1; **j**–**l**) obtained from 6-month-old 14-3-3eta^WT/WT^ (**a**, **d**, **g** and **j**), 14-3-3eta^GT/WT^ (**b**, **e**, **h** and **k**) and 14-3-3eta^GT/GT^ (**c**, **f**, **i** and **l**) mice. Both IHCs and OHCs are regularly aligned in the apical and middle turn of all genotype. In the basal turn (B1, B2) OHCs loss is observed in 14-3-3eta^GT/WT^ and 14-3-3eta^GT/GT^. Scale bars: 5 *μ*m.

**Figure 3:**
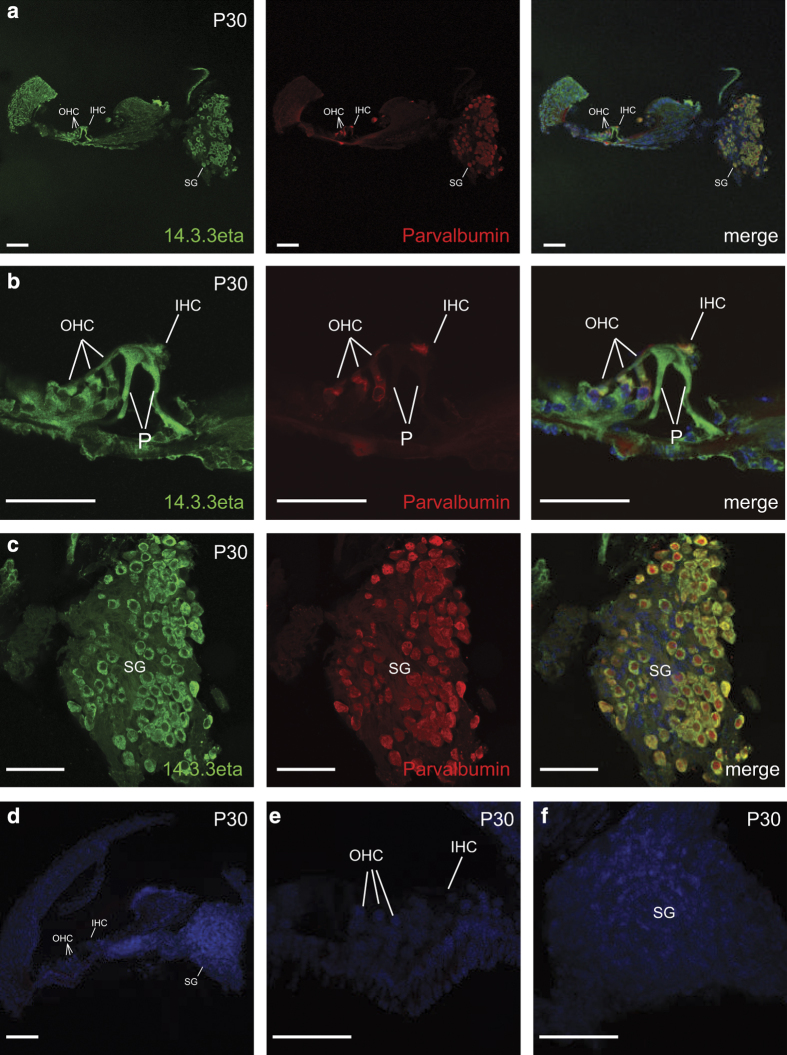
14-3-3eta expression in cochlea. Microdissected adult (P30) mice cochlea were stained with Hoechst and immuno-labeled with an anti-14-3-3eta antibody (in green) and antiparvalbumin antibody (in red) to identify hair cells and spiral ganglion neurons. (**a**) Strong expression of 14-3-3eta was localized in inner (IHC) and OHCs, pillar cells (P) and spiral ganglion (SG). (**b**) Higher magnification image of organ of Corti showing staining of hair cells and pillar cells. (**c**) Higher magnification image of spiral ganglion neurons. (**d**) Control section of cochlea. (**e**) Control section of the organ of Corti. (**f**) Control section of spiral ganglion neurons showing no staining. Scale bar represents 100 *μ*m.

As cochlear function was impaired, we sought for histological alterations. Cochleas from 6-month-old 14-3-3eta^GT/WT^, 14-3-3eta^GT/GT^ and 14-3-3eta^WT/WT^ mice were examined by scanning electron microscopy (Figure 2). Different parts of the cochlea were observed: the apex (A), the midturn (M), and the two most basal turns (B1 and B2). In the midturn and apical parts of the cochlea, the structure of the organ of Corti was normal in 14-3-3eta ^GT/WT^ (Figures 2b and e) and 14-3-3eta^GT/GT^ (Figures 2c and f) mice, the single row of inner hair cell (IHC) and the three rows of OHCs being preserved as in 14-3-3eta^WT/WT^ mice (Figures 2a and d). In contrast, a prominent finding observed in the 14-3-3eta^GT/WT^ (Figures 2h and k) and 14-3-3eta^GT/GT^ (Figures 2i and l) mice was the abrupt occurrence of massive OHC loss in the basal part of the cochlea compared with 14-3-3eta^WT/WT^ (Figures 2g and j). In addition, some IHCs were missing in B2 in 14-3-3eta^GT/GT^ (Figure 2i).

**Figure 4:**
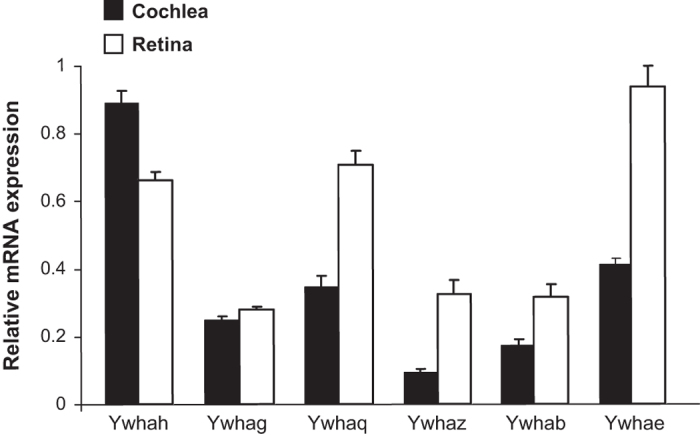
Expression of 14-3-3 transcripts in mouse retina and cochlea. Relative mRNA expression of 14-3-3 transcripts in mouse cochlea and retina were quantified by qPCR. Data are mean±S.E.M.

We then examined the tissue expression of 14-3-3eta in the sensory organs. In the cochlea, 14-3-3eta was strongly expressed in the IHC and OHC and in the pillar cells of the organ of Corti (Figures 3a and b). The spiral ganglion neurons were also strongly labeled (Figures 3a and c), whereas no signal was detected in negative controls (Figures 3d–f). In the retina, 14-3-3eta was highly expressed in RGC and slightly in neurons of the inner nuclear layer but not in photoreceptors (Supplementary Figure S3). We then measured the expression level of all 14-3-3 isoform transcripts in adult mice by quantitative PCR. In the cochlea, 14-3-3eta had the highest expression, being at least twofold more abundant than any other 14-3-3 isoform (Figure 4). In the retina, 14-3-3eta was also highly expressed, with a level similar to that of the theta and epsilon isoforms (Figure 4). Altogether, 14-3-3 proteins were expressed at a higher level in the cochlea than in the retina, with the highest level for 14-3-3eta.

### *Ywhah* loss of function induces apoptosis in mouse cochlea

**Figure 5:**
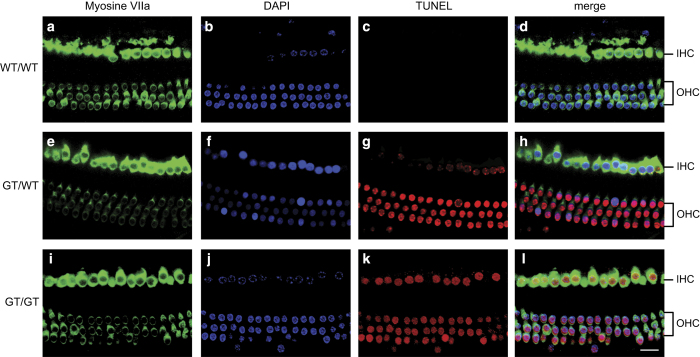
Detection of apoptosis by TUNEL assay in control and mutant cochlea. (**a**, **e**, **i**) Wild-type and mutant organ of Corti immunostained for Myosine VIIa (green) to detect hair cells. (**b**, **f**, **j**) DAPI (blue) and (**c**, **g**, **k**) TUNEL (red) staining observed in the organ of Corti in basal turn for 14-3-3eta^WT/WT^, 14-3-3eta^GT/WT^ and 14-3-3eta^GT/GT^. (**c**) No TUNEL-positive cells were detected in the control cochlea. (**g**, **k**) Positive TUNEL staining was seen in both 14-3-3eta^GT/WT^ and 14-3-3eta^GT/GT^. (**d**, **h**, **l**) Merge images with Myosine VIIa (green), DAPI (blue) and TUNEL (red). Scale bar=20 *μ*m.

To determine whether the inner ear cell loss was due to apoptosis, we performed TUNEL (terminal deoxunucleotidyl transferase biotin-dUTP nick end labeling) staining to examine cochleas from 6-month-old 14-3-3eta^WT/WT^, 14-3-3eta^GT/WT^ and 14-3-3eta^GT/GT^ mice (Figure 5). Whereas no TUNEL-positive cells were detected in 14-3-3eta^WT/WT^ cochleas, apoptosis was clearly seen in OHCs and IHCs of the basal part of cochleas from 14-3-3eta^GT/WT^ and 14-3-3eta^GT/GT^ (Figure 5). These observations support the notion that apoptosis takes place in response to loss of 14-3-3eta and can be the mechanism responsible for cell death in mutant cochleas. These results provide evidence that *Ywhah* loss of function results in induction of apoptosis in the cochlea.

### Identification of novel *YWHAH* non-synonymous variants in deaf population

**Figure 6:**
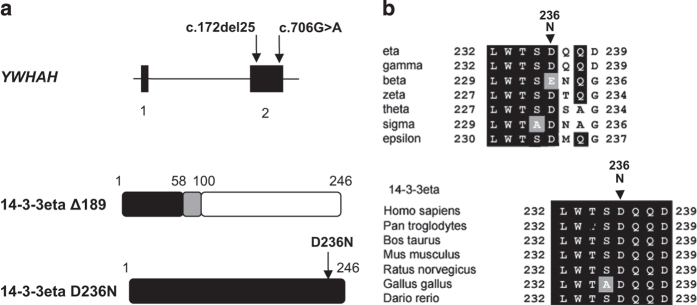
Identification of damaging variants in *YWHAH* gene. (**a**) Intron–exon structure of the *YWHAH* gene and position of variants in exon 2 (top). From top to bottom, schematic representation of the 14-3-3eta protein, position of variants and consequence of the deletion in protein translation (the gray box represents the frameshift, dotted lines represent truncative part of the protein). (**b**) Amino-acid alignment of the C-terminal domain of different 14-3-3 isoforms (top) and of 14-3-3eta from different species (bottom) showing the conserved aspartate at position 236. Conserved, semi-conserved and non-conserved substitutions are framed in black, gray and white boxes, respectively.

Knockout mice and tissue expression results suggested that 14-3-3eta could, when mutated or dysregulated, lead to auditory impairment in humans. We thus screened the *YWHAH* gene (Genbank accession number NM_003405.3) composed of two exons separated by an 8-kb intron^20^ in 793 unrelated patients with non-syndromic or syndromic deafness. We found one frameshift, six missense and one intronic heterozygous SNP (single-nucleotide polymorphism), which were absent from the dbSNP, HGVD (Human Genetic Variation Database) databases, Exome Variant Server and 1000 genomes database (Table 1). Two of them, c.172del25 and c.706G>A, were predicted to be damaging or deleterious. The c.172del25 (p.Ser58Leufs*43) was predicted to produce a truncated 14-3-3eta protein with the loss of 60% of protein sequence, including the substrate-binding domain^4^ (Figure 6a). The c.706G>A (p.Asp236Asn) changes an amino acid involved in target binding and highly conserved across species and among 14-3-3 isoforms^4^ (Figure 6b). The other variants were predicted to be either possibly damaging or benign (Table 1).

### *YWHAH* non-synonymous variants increase apoptosis and abolish Bad interaction

**Figure 7:**
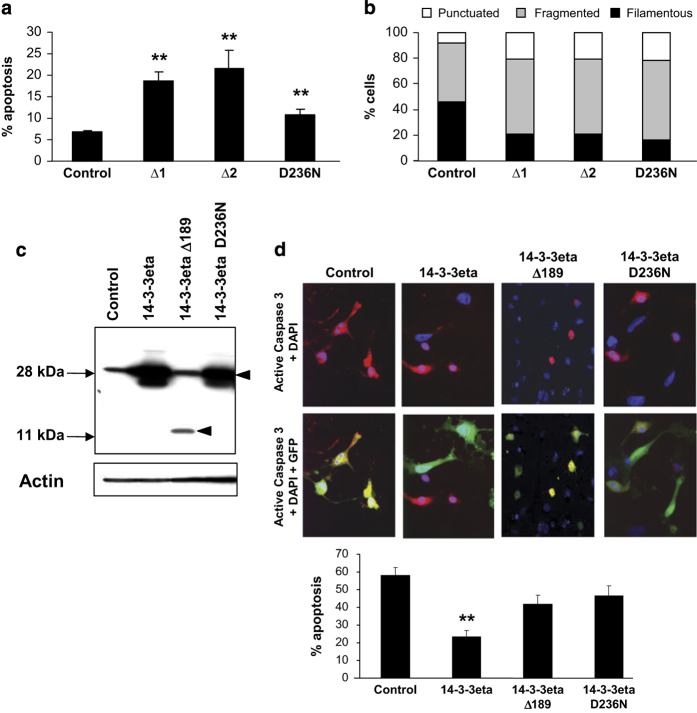
Impact of *YWHAH* variants in human fibroblasts. Fibroblasts from patients (family 1: the father ∆1 and his son ∆2, family 2: D236N) were compared in all experiments with three control fibroblast cultures obtained from healthy patients. (**a**) Susceptibility to apoptosis was assessed by counting apoptotic nuclei after a 3-h staurosporine treatment (1 *μ*M; *n*=3). (**b**) Mitochondrial network structure was assessed on exponential growing cultures after Mitotracker staining. Filamentous, fragmented and punctuated phenotypes were counted on 200 cells in 3 independent experiments. Data are mean±S.E.M. (**c**) HeLa cells were transiently transfected with pIRES-EGFP vectors expressing 14-3-3eta, 14-3-3eta ∆189 and 14-3-3eta D236N. Western blotting of total cell lysate showing the expression of the different proteins (arrows). Actin was used as a loading control. (**d**) In a parallel experiment, HeLa cells were treated with 1 *μ*M staurosporine for 3 h before the quantification of apoptotic cells. Apoptosis was assessed by immunofluorescent detection (top) of the active form of caspase*-*3 (red fluorescence) in transfected cells (green fluorescence). All cells were stained with the nuclear marker Hoechst. Scale bar represents 20 *μ*m. Quantification of apoptosis in transfected cells (bottom), each point representing the average of triplicate±S.E.M.

As c.172del25 and c.706G>A variants would produce 14-3-3eta proteins with a modified target-binding site, we studied skin fibroblasts carrying these variants. Considering the role of 14-3-3eta in cell death, we analyzed the susceptibility to apoptosis in patient fibroblasts. We found a marked increase in staurosporine-induced cell death (Figure 7a), which was more severe in the c.172del25 (Δ) mutated cells as compared with c.706G>A (Asp236Asn) fibroblasts and a higher fragmentation of the mitochondrial network in patient fibroblasts compared with controls (Figure 7b).

To confirm these observations, we expressed the wild-type and mutated 14-3-3eta isoforms in HeLa cells to monitor their protective effect on staurosporine-induced apoptosis. Vectors expressing 14-3-3eta, 14-3-3eta ∆189 and 14-3-3eta D236N were transiently transfected in HeLa cells (Figure 7c). Twenty-four hours after transfection, staurosporine was added to HeLa cells. Wild-type 14-3-3eta provided strong inhibition of apoptosis (*P*=0.0046), whereas the mutated forms failed to significantly protect from apoptosis (Figure 7d), showing that variants in *YWHAH* altered the antiapoptotic function of 14-3-3eta. Overexpression of wild-type or mutated 14-3-3eta remained without effect on the mitochondrial network and on the mitochondrial membrane potential.

**Figure 8:**
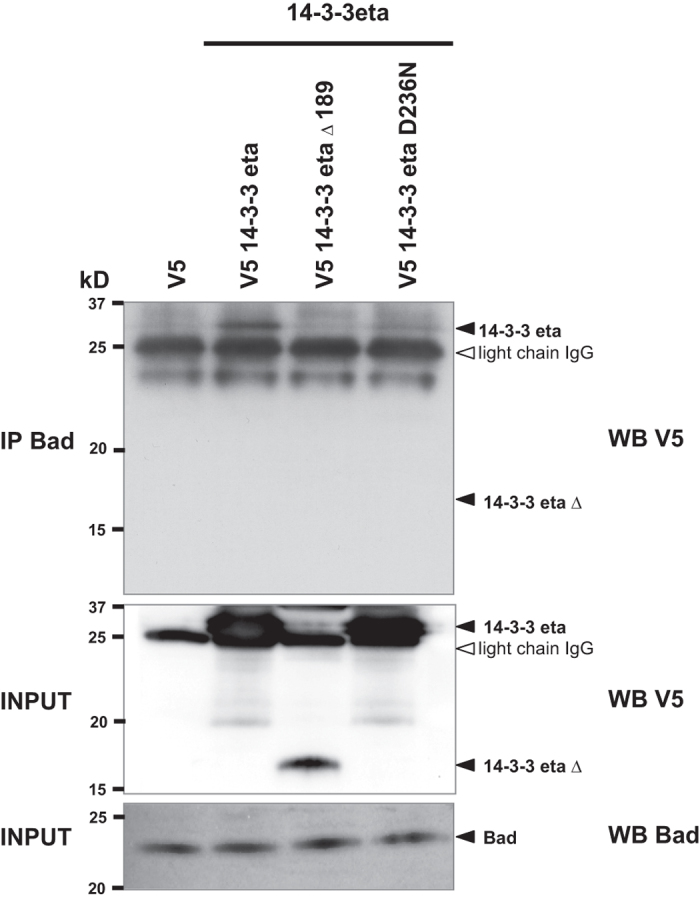
Impact of *YWHAH* variants on Bad interaction. After transfection of empty vector pcDNA-V5, 14-3-3eta-V5, 14-3-3eta∆189-V5 and 14-3-3D236N-V5 in HeLa cells, the production of the protein was verified by western blotting (Input, middle panel), and immunoprecipitation studies were performed with the Bad antibody. The presence of 14-3-3eta in the Bad complex was shown by immunoblot with the rabbit anti-V5 antibody (IP Bad, top panel). The antibody used for the immunoblot analysis is denoted at the right side. Cell lysates were immunoblotted with the Bad antibody as a control (Input, bottom panel).

Finally, to obtain further insight into the antiapoptotic function of mutated 14-3-3eta, we analyzed the interaction of 14-3-3eta with Bad, a pro-apoptotic member of the Bcl-2 family^21^ by immunoprecipitation. 14-3-3 binds and retains phosphorylated Bad in the cytoplasm. When dissociated from 14-3-3, Bad translocates to mitochondria where it binds and inactivates the pro-survival proteins Bcl-2/Bcl-xL, leading to initiation of the apoptotic cell death process.^22^ We found that the wild-type 14-3-3eta co-immunoprecipitated with Bad, whereas both variants abolished Bad binding (Figure 8), confirming that 14-3-3eta C-terminal domain was involved in Bad interaction and that consequently alterations in this domain facilitate Bad translocation from the cytoplasm to the mitochondrial outer membrane, thus promoting excessive apoptosis.

## Discussion

In this study, we showed that the absence of 14-3-3eta causes a hearing loss with a cochlear OHCs' degeneration in mice, only for high frequencies, that is, from 30 to 90 kHz. We found that 14-3-3 proteins are expressed in both cochlea and retina, at various levels, with the highest expression of 14-3-3eta in the cochlea. In humans, we identified two variants at the heterozygous state in *YWHAH*, never described before, and we demonstrated that they indeed abolished the protein function.

The auditory exploration of the knockout mouse revealed an early and non-progressing decrease of the ABRs, suggesting a congenital impairment of hearing in 14-3-3eta^GT/WT^ and 14-3-3eta^GT/GT^ mice. However, we have not observed cell loss in the cochlea areas corresponding to the frequencies tested, which represent the median and apical region of the cochlea. Little is known about the role of 14-3-3 proteins in the inner ear. We demonstrated that loss of hair cells in the basal part of 14-3-3eta mutant cochleas are related to apoptosis and highlight the role of 14-3-3eta in OHC survival. These results were consistent with the notion that apoptosis is a major cause of hearing loss in mammals.^23,24^ Additionally, Bcl-2 family proteins such as Bax and Bad are critical for the maintenance of hearing function.^25^ Further data suggest a critical role of this apoptotic pathway in IHCs' survival, as mutations in the pro-apoptotic protein SMAC/DIABLO were reported in human dominant hearing loss DFNA64^26^ and the pro-apoptotic Bak protein is involved in age-related hearing loss in mice.^27^ In the cochlea, death of OHCs after an acoustic trauma has been linked to the activation of pro-apoptotic Bad.^28^ Altogether, these studies suggest that Bad is a crucial determinant of cell fate in the cochlea. Therefore, the defects caused by 14-3-3eta dysfunction might result in Bad activation leading to or facilitating the degeneration of cells that specifically rely on high *YWHAH* expression.

Surprisingly, the absence of 14-3-3eta did not cause electrophysiological and histological alterations of the retina, whereas because of the abundant 14-3-3eta expression in the RGCs, we expected visual impairment. We noted that there was no significant increase in the expression levels of other 14-3-3 in 14-3-3eta-deficient mice (data not shown), therefore there is no clues for a compensatory upregulation of other 14-3-3 isoforms in the retina. Yet, it is still possible that the endogenous levels of other 14-3-3 isoforms were sufficient to compensate for the 14-3-3eta loss, especially in the retina where other 14-3-3 are predominant. In contrast, 14-3-3eta is the predominant isoform in the cochlea, therefore its deficiency may not be sufficiently compensated by other isoforms.

Considering the impact of the loss of 14-3-3eta on the survival of hair cells, we investigated whether this protein could lead to auditory diseases. *YWHAH* has been extensively screened in large cohorts of patients (>2000 cases) and controls (>1200 cases) because the *YWHAH* chromosomal location at 22q12.3 co-localizes with the susceptibility loci identified in schizophrenia,^29,30,31,32^ bipolar disorders^33^ and Parkinson disease.^34^ However, non-synonymous variants were never identified. By screening *YWHAH* gene in non-syndromic and syndromic deafness, we reported seven non-synonymous variations never previously found in this gene. We wanted to test whether these variants could impair the 14-3-3eta function. To define the biological function of 14-3-3eta, we have used a combination of two models: downregulation of 14-3-3eta with patient fibroblasts that act for a condition of haploinsufficiency and overexpression of 14-3-3eta mutants in wild-type HeLa cells. We showed that these variants confer an increased susceptibility to apoptosis in fibroblasts, which we were able to reproduce by expressing 14-3-3eta variants in a heterologous system. This deregulated cell death control was related to impair Bad binding to 14-3-3eta. This latter observation further correlates with the alteration of the mitochondrial network fusion process observed in fibroblasts, which is known to be controlled by pro-apoptotic and antiapoptotic members of the Bcl2 family. As 14-3-3 molecules can interact with a wide variety of cellular proteins, it is possible that genetic variants affect the protection against apoptosis.

This study shows the essential role of 14-3-3eta in OHC survival. In addition, we report for the first time *YWHAH* variants that can significantly alter the antiapoptotic function of 14-3-3eta by abolishing its interaction with Bad. Altogether, our results underline the fundamental role of 14-3-3eta in auditory physiology.

## Materials and Methods

### Examination of mouse electrophysiological audition

#### Animal surgery

All procedures were performed under general anesthesia achieved by an intra-peritoneal injection of a mixture of Rompun 2% (3 mg/kg) and Zoletyl 50 (40 mg/kg). For DPOAEs and for endocochlear potential recordings, both pinna were severed. Moreover, for endocochlear potential recordings, a retro auricular incision was performed and the bulla tympani was opened to expose the cochlea.

#### Auditory brain stem recordings

The acoustical stimuli were produced by an arbitrary function generator (type 9100 R; Le Croy Corporation, Chesnut Ridge, NY, USA). They consisted in 3-ms tone pips with a 1-ms rise and fall time at a rate of 10/s. Tone pips were passed through a programmable attenuator and delivered to the animal by a JBL 075 loudspeaker (James B. Lansing Sound, Los Angeles, CA, USA) in a calibrated free field. ABRs were differentially derived with subcutaneous needle electrodes placed at the vertex and laterally below the stimulated auditory canal with a reference below the controlateral auditory canal. The signal was amplified (×20 000) (P 511 K, Grass Technologies, West Warwick, RI, USA), digitalized (50 kHz sampling rate, with a 12-bit dynamic range analog to digital converter), averaged 900 times and stored on a computer (Dell Dimention, Austin, TX, USA). Recordings were made at 2, 4, 6, 8, 10, 12, 16, 20, 26 and 32 kHz, and intensity was decreased from 100 to 0 dB sound pressure level (SPL)) (reference 2.10–5 Pa) by 5-dB steps. The threshold was determined as the lowest intensity able to elicit a visible ABR.

#### Distortion product oto-acoustic emission recordings

DPOAEs were recorded in the external auditory canal with an ER-10C S/N probe (Etymotic Research, Elk Grove Village, IL, USA) consisting of two emitters and one microphone. The two primary tones were generated, and the distortion was processed by the Cubdis system HID 40133DP (Mimosa Acoustics, Champaign, IL, USA). The probe was self-calibrated for the two stimulating tones before each recording. The two tones were presented simultaneously. *F*_2_ was swept from 0.5 to 20 kHz by quarter of octave steps and the *f*_1_/*f*_2_ ratio was maintained constant at 1.2. The intensities of *f*_1_ and *f*_2_ were set at 60 and 55 dB SPL, respectively. For each frequency, the cubic distortion product (2*f*_1_−*f*_2_) and the neighboring noise magnitude were measured and expressed as a function of *f*_2_.

#### Endocochlear potential recordings

After opening the bula tympani, a small hole was made in the otic capsule over the stria vascularis of the basal turn by gently eroding the bone with a custom-designed blade. A glass micropipette filled with 3 M KCl was inserted in the scala media through the stria vascularis with a micromanipulator (Warner Instruments, CT, USA). This electrode, together with a reference needle electrode placed in the posterior paw was connected to a DC amplifier (KS-700, WPI, Sarasota, FL, USA) though an impedance adaptor. The endocochlear potential value was directly read on the amplifier display.

### qRT-PCR

Total RNA was extracted from different organs of adult mice using the RNeasyMini Kit (Qiagen, Venlo, The Netherlands) and Dnase-treated with RQ1 DNase (Promega, Madison, WI, USA) according to the manufacturer’s instructions. All product lengths were restricted between 100 and 150 pb to allow the highest efficiency of amplification. In all, 1 *μ*g of RNA product was reverse-transcribed with 200 U of Superscript III reverse transcriptase (Invitrogen, Carlsbad, CA, USA) and 250 ng hexamer random primers (Promega). For quantitative RealTime PCR, LightCycler Fast Start DNA MasterPlusSYBR GreenI ready-to-use reaction mix (Roche Diagnostics, Meylan, France) was used following the manufacturer's instructions. Primer pairs (Supplementary Figure S4a) were designed and optimized to ensure specific amplification of the target sequences.

### Immunohistochemistry

Eyes and cochleas were fixed in cold 4% paraformaldehyde overnight. After tissue cryoprotection and inclusion, 12-*μ*m sagittal sections were collected in the mid-retina, at the optic nerve head,^35^ and 14 *μ*m were taken throughout the entire cochlear.^36^ Frozen sections were stained with Hoescht (1/1 000, Invitrogen), 14-3-3eta antibody (1/500, Millipore, Molsheim, France) and parvalbumin (1 : 750, Swants, Marly, Switzerland). Microscope slides were visualized using a Zeiss LSM 510 Meta confocal microscope (Marly le Roi, France).

### TUNEL assay on cochlea

Six-month-old mice were euthanized by intracardiac perfusion with 4% PFA. Cochleas were micro-dissected in phosphate-buffered saline (PBS). Cochleas were immunostained for Myosine VIIa (Proteus Biosciences, Ramona, USA) and evaluated for TUNEL labeling using the *In S* *itu* Cell Death Detection Kit (Roche Diagnostics) according to the manufacturer’s protocol. Cell nuclei were stained with DAPI. Cochleas were then mounted on a slide, coverslipped and imaged using a Zeiss LSM 510 Meta confocal microscope.

### Immunoprecipitation

For immunoprecipitation experiments, mutant and wild-type14-3-3eta cDNAs were cloned into pcDNA/V5-His vector and transfected in Hela cells using Lipofectamine 2 000 (Invitrogen). Twenty-four hours after transfection, cells were lysed with 1% Triton lysis buffer (100 mM NaCl, 1% Triton, 0.02 M Tris, 0.005 EDTA) at 4 °C for 1 h. Mouse anti-Bad antibody (BD Transduction Laboratories, Franklin Lakes, NJ, USA) was previously coupled to protein-G Sepharose and used for immunoprecipitation. The presence of 14-3-3eta in the Bad complex was revealed by immunoblot with the rabbit anti-V5 antibody (Invitrogen).

### *YWHAH* screening

Genomic DNA was extracted from blood samples by standard methods. A hundred nanograms of DNA was amplified with Taq DNA polymerase (AmpliTaq Gold, Applied BioSystems, Foster City, CA, USA) helped with GC-RICH resolution solution (Roche Diagnostics) for exon 1, using intronic primers representing each exon (Supplementary Figure S4b). Standard cycling conditions were carried out with optimized annealing temperatures: 30 s at 95 °C, 1 min at 65 °C for exon 1 and 30 s at 60 °C for exon 2, and 1 min at 72 °C for the 35 cycles, followed by a final elongation at 72 °C for 5 min. PCR products were cycled-sequenced using BigDye terminator system (Applied BioSystems), and analyzed on an ABI Prism 3130 capillary sequencer (Applied BioSystems).

### Patients

The cohort for *YWHAH* screening consisted of 793 unrelated patients with syndromic or non-syndromic deafness seen in different Medical Centers (France, Belgium, United Kingdom, Marroco). These patients were referred for genetic deafness. The study was approved by all institutional review boards of the participating institutions, and written informed consent was obtained from all participants or their legal guardians. Patients with non-syndromic deafness were negative for *GJB2* and *GJB6* mutations, which are most prevalent genes causing autosomal-recessive non-syndromic deafness.

### Patient’s fibroblasts

Fibroblast cultures were obtained from a skin biopsy as previously described.^37^ Apoptosis susceptibility, mitochondrial network structure, JC1 labeling and respiratory parameters were performed as described previously.^38,39^

### Vector construction

Mammalian expression vectors for 14-3-3eta, 14-3-3eta Δ189 and 14-3-3eta D236N were constructed by PCR amplification of the corresponding human cDNA fragment and subsequently cloned into CMV promoter-based expression vectors pIRES-EGFP (Clontech, Mountain View, CA, USA) or pcDNA/V5-His vector (Invitrogen). Empty vector pIRES-EGFP and pcDNA/V5-His were used as control.

### Cell culture and transfection

HeLa cells were cultured in complete culture medium (Dulbecco's modified Eagle's medium, supplemented with 10% fetal calf serum and 100 units/ml penicillin/streptomycin, Invitrogen) and maintained at 37 °C in a humidified 5% CO_2_ atmosphere. Cells were seeded at a concentration of a 2×10^5^ into six-well plates. Transient transfections were performed using Lipofectamine 2000 in Optimem (Invitrogen) according to the manufacturer's protocol. Twenty-four hours after transfection, staurosporine (Sigma, St. Louis, MO, USA; 1 *μ*m for 3 h) was added and then apoptotic nuclei stained with Hoechst (Invitrogen) and counted in EGFP-expressing cells (*n*=200) or by immunofluorescence using a cleaved caspase-3 antibody (1/10, Millipore). Mitochondrial network was evaluated by immunostaining of mitochondria with ATP synthase antibodies (1/500, Millipore). Cells were stained with JC1 (Invitrogen) to analyze mitochondrial membrane potential.^38^

### Scanning electron microscopy

The mice were decapitated under deep anesthesia (Pentobarbital 50 mg/kg), and their cochleae were removed from the temporal bone and fixed in 2.5% glutaraldehyde in 0.1 M–pH 7.3 PBS. The otic capsule was dissected out, and the stria vascularis, tectorial and Reissner's membranes removed. After rinsing in PBS (pH 7.3), the samples were dehydrated in a graded series of ethanol (30–100%), critical point dried in CO_2_, coated with gold palladium and observed using a Hitachi S4000 microscope (Krefeld, Germany).

### Western blot

Cells and tissues were lysed in total extraction buffer containing 10 mM Tris-HCl pH 6.8, 1 mM EDTA and 1% SDS prewarmed at 95 °C. Protein content was quantified by a modified Lowry assay (BioRad DC protein assay, Bio-Rad, Hercules, CA, USA). In all, 50 *μ*g of protein were electrophoresed on 15% SDS-polyacrylamide gels and transferred to nitrocellulose. Immunodetection was performed with the ECL (Electrochemiluminescence) Western blotting analysis system (GE Healthcare, Buckinghamshire, UK) Antibodies used were anti-14-3-3eta antibody (1/3000, Millipore), actin antibody (1/5000, Sigma) and tubulin antibody (1/5000, Sigma).

### Statistical analyses

Values given represent means±S.D. or means±S.E.M. Statistical significance was determined at the *P*<0.05 (*), *P*<0.01 (**) and *P*<0.001 (***) levels using a Student's two-tailed unpaired *t*-test.

## Figures and Tables

**Table 1:** *YWHAH* variants detected in non-syndromic and syndromic deafness

*Family*	*Nucleotide change*	*Amino-acid change*	*Status*	*Localization*	*Phenotype*	*Polyphen2 (score)*	*SIFT (score)*	*Provean (score)*
1	c.172del25	p.Ser58Leufs*43	Heterozygous	Exon 2	Deafness+OA	NA	NA	Deleterious (−139.3)
2	c.706G>A	p.Asp236Asn	Heterozygous	Exon 2	Deafness+OA	Probably damaging (0.998)	Affect protein function(0.0)	Deleterious (0.998)
3	c.722A>C	p.Glu241Ala	Heterozygous	Exon 2	Familial deafness	Possibly damaging (0.698)	Affect protein function (0.01)	Neutral
4	IVS1+67 C>T	No change	Heterozygous	Intron 1	Familial deafness	NA	NA	NA
5	c.104A>G	p.Glu35Gly	Heterozygous	Exon 2	Familial deafness	Possibly damaging (0.552)	Affect protein function (0.0)	Neutral
6	c.187A>G	p.Ile63Val	Heterozygous	Exon 2	Sporadic deafness	Benign	Affect protein function (0.05)	Neutral
7	c.295A>G	p.Asn98Ser	Heterozygous	Exon 2	Sporadic deafness	Benign	Affect protein function (0.02)	Neutral
8	c.338A>G	p.Asn113Ser	Heterozygous	Exon 2	Sporadic deafness	Benign	Tolerated (1.00)	Neutral

Abbreviation: NA, not applicable.
