# Supplementary figures and images for: Loss of function of Ywhah in mice induces deafness and cochlear outer hair cells' degeneration
Source: Cell Death Discov. 2016 Mar 7;2:16017–. doi: 10.1038/cddiscovery.2016.17 (PMC4893315; doi:10.1038/cddiscovery.2016.17)

**a**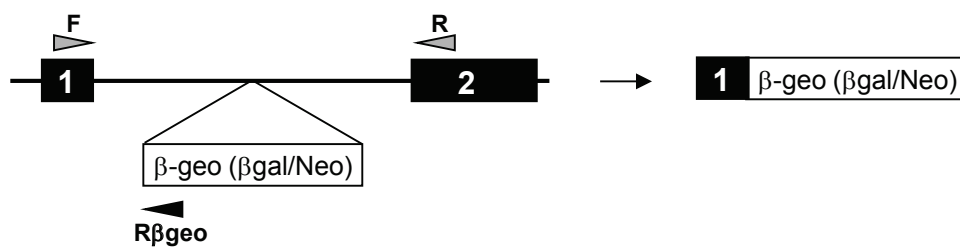**b**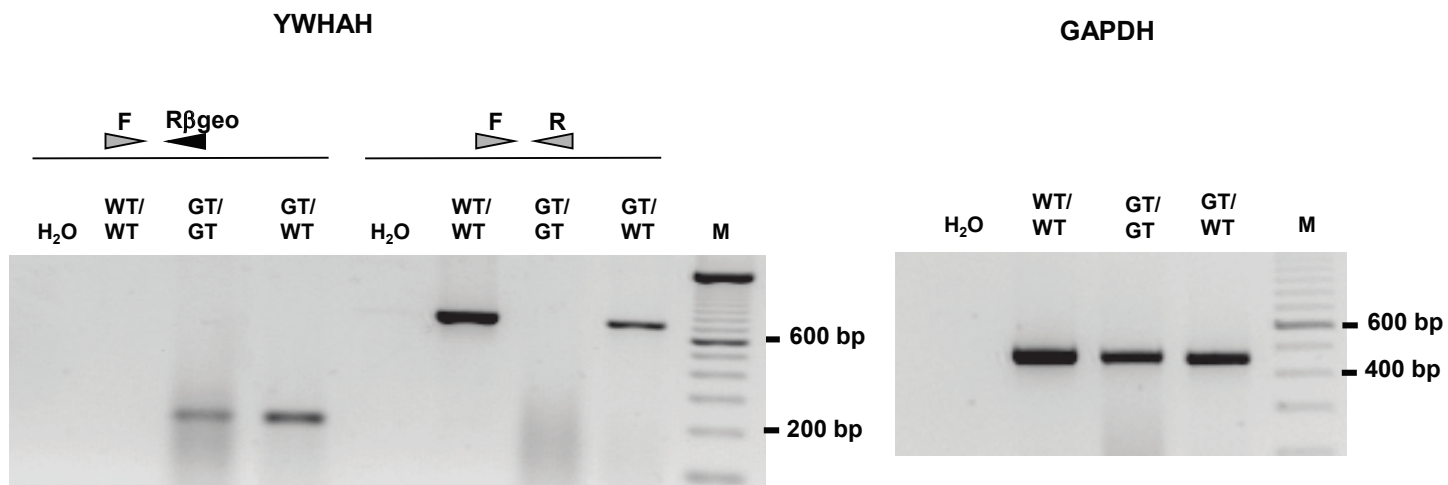**c**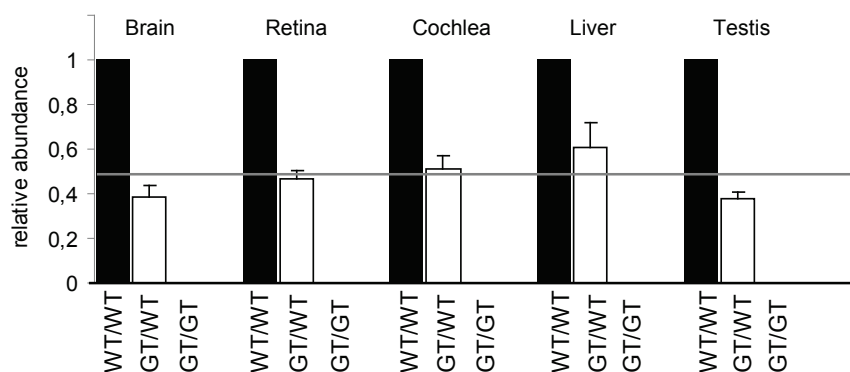**d**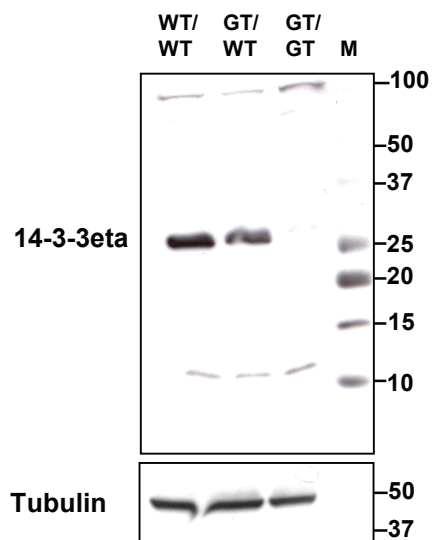

Supplement: Supplementary Figure S1 [file cddiscovery201617-s1.pdf]

**a**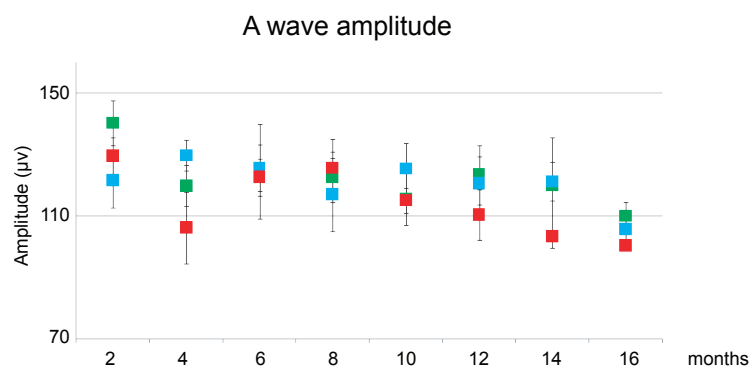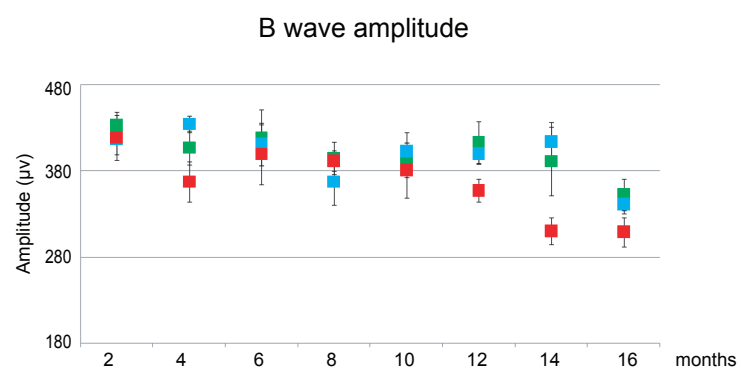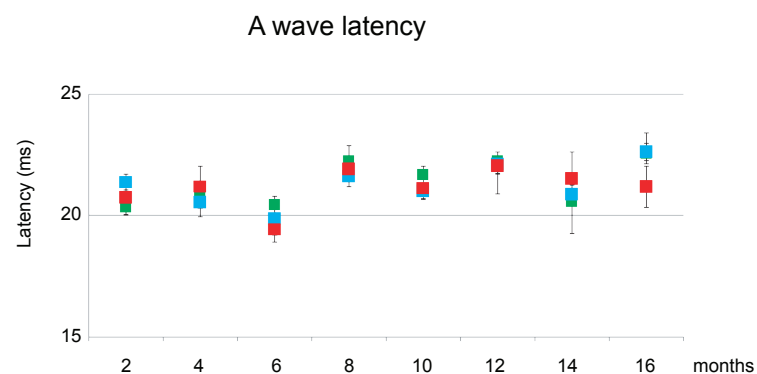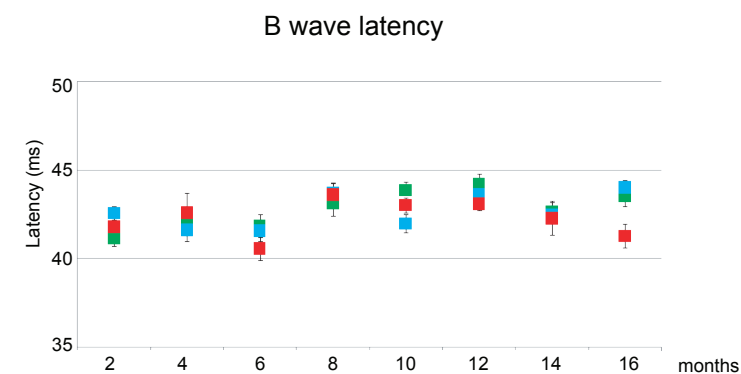**b**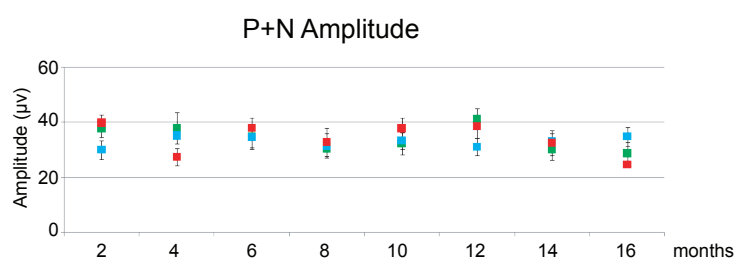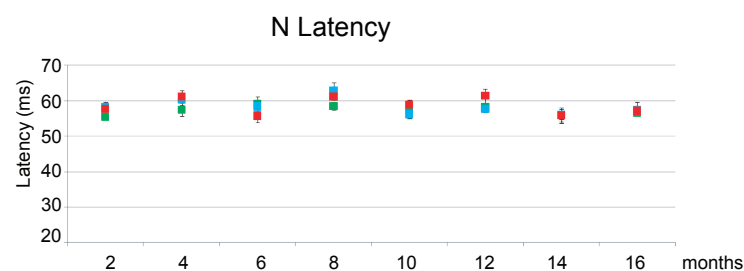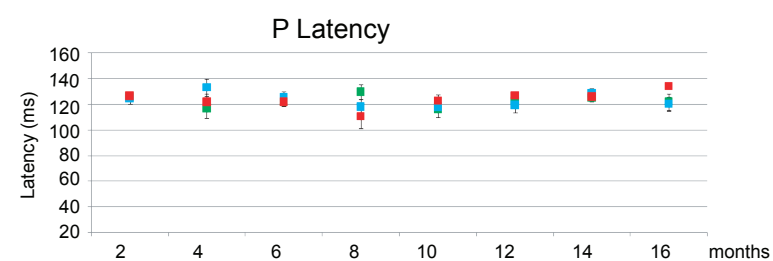**c**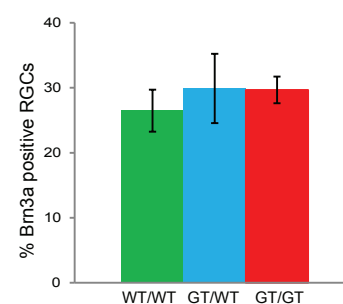

Supplement: Supplementary Figure S2 [file cddiscovery201617-s2.pdf]

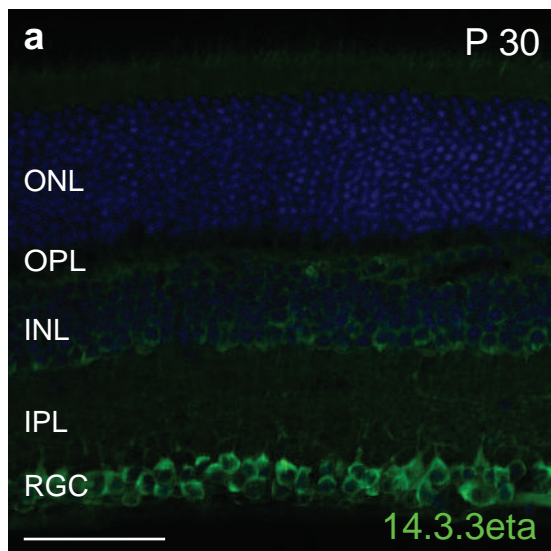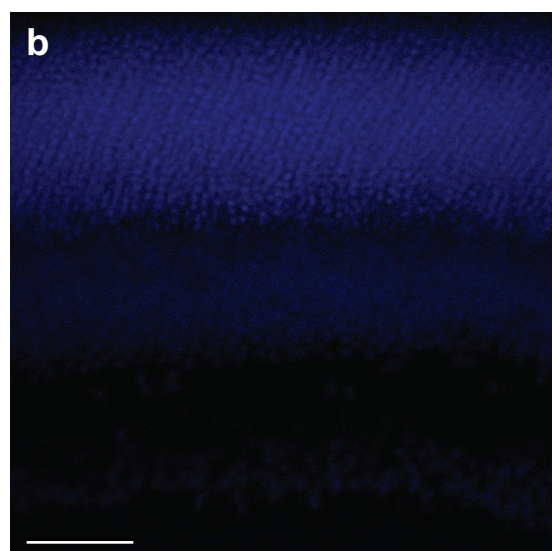

Supplement: Supplementary Figure S3 [file cddiscovery201617-s3.pdf]
