# Supplementary material for: Loss of function of Ywhah in mice induces deafness and cochlear outer hair cells' degeneration
Source: Cell Death Discov. 2016 Mar 7;2:16017–. doi: 10.1038/cddiscovery.2016.17 (PMC4893315; doi:10.1038/cddiscovery.2016.17)
Supplement: Supplementary Figure S4 [file cddiscovery201617-s4.doc]

**a**

Mouse primer sequences used for quantitative RT-PCR

| Primer Name | Sequence 5’-3’ |
| --- | --- |
| Ywhah-F | CTTAGCCAAACAAGCCTTCG |
| Ywhah-R | ATCTGAATAGCTGTGCTGCC |
|  |  |
| Ywhag-F | AGCAACTGGTGCAGAAAGC |
| Ywhag-R | TTGGACAGTGGTTCGTTCAG |
|  |  |
| Ywhaq-F | ATCCAGAGCTTGCCTGCACA |
| Ywhaq-R | AAGCAACTGCATGATGAGGGT |
|  |  |
| Ywhaz-F | CACAGCAAGCATACCAAGAA |
| Ywhaz-R | AGAATGAGGCAGACAAAGGT |
|  |  |
| Ywhab-F | CCAGCAGGCTTACCAAGAAG |
| Ywhab-R | CAGGCCTTTTCAGGAGAGTTT |
|  |  |
| Ywhae-F | TAACACTGGCGAGTCCAAGGTT |
| Ywhae-R | GCGTTGGAGGAAGTTCTGTC |
|  |  |
| L27-F | ACGCAAAGCCGTCATCGTGAAG |
| L27-R | CTTGGCGATCTTCTTCTTGCC |

**b**

Human primer sequences used for *YWHAH* screening

| Primer Name | Sequence 5’-3’ |
| --- | --- |
| EX1-F | GGCGAGCCAGTGCGCGTGCG |
| EX1-R | GTCGCCGGGTCGCCCAT |
|  |  |
| EX2-F | CCGTTCTTGAGAAGGATTGTTG |
| EX2-R | GCAAGGAAGAATCGGTGATGA |
