# Supplementary material for: Loss of function of Ywhah in mice induces deafness and cochlear outer hair cells' degeneration
Source: Cell Death Discov. 2016 Mar 7;2:16017–. doi: 10.1038/cddiscovery.2016.17 (PMC4893315; doi:10.1038/cddiscovery.2016.17)
Supplement: Supplementary Figure Legends [file cddiscovery201617-s5.doc]

**Supplementary Figure Legends**

**Supplementary Figure S1.** Production and validation of Ywhah deficient mice. (**a**) Diagram (not to scale) of genomic structure of *Ywhah* and gene-trap insert in intron 1. β-geo, fusion of β-galactosidase and neomycin transferase. Arrowheads, approximate location of PCR primers used to verify the gene trap insertion on mRNA. (**b**) RT-PCR analysis showing reduced amount of Ywhah mRNA in 14-3-3eta^GT/WT^ mice and its absence 14-3-3eta^GT/GT^ mice. Arrowheads, primers shown in A. GAPDH mRNA was used as control. (**c**) qRT-PCR analysis confirming reduction of Ywhah in different tissues. Data are mean ± SEM. (**d**) Protein blot analysis with anti-14-3-3eta antibody showing reduced amount of the 28 kDa 14-3-3eta protein in GT/WT mice and no protein in GT/GT mice. α-Tubulin was probed as loading control

**Supplementary Figure S2.** Visual function of 14-3-3eta mutant mice. (**a**) Electroretinograms were performed on mice from 2 months to 16 months. Mean ERG amplitudes and latencies of A wave (left) and mean ERG amplitudes and latencies of B wave (right) in 14-3-3eta^WT/WT^ (green) (6≤n≤10), 14-3-3eta^GT/WT^ (blue) (9≤n≤13) and 14-3-3eta^GT/GT^ (red) (6≤n≤12) for a stimulation of 159 cd.s^-1^.m^-2^. Data are mean ± SEM. (**b**) Visual evoked potentials (VEP) on mice from 2 months to 16 months. Mean of VEP amplitudes of N+P waves in 14-3-3eta^WT/WT^ (green) (5≤n≤9), 14-3-3eta^GT/WT^ (blue) (6≤n≤11) and 14-3-3eta^GT/GT^ (red) (4≤n≤9) for a stimulation of 159 cd.s^-1^.m^-2^. Data are mean ± SEM. (**c**) Percentage of retinal ganglion cells positive for Brn3a in 12 months retinas in 14-3-3eta^WT/WT^ (green), 14-3-3eta^GT/WT^ (blue) and 14-3-3eta^GT/GT^ (red). Data are mean ± SEM

**Supplementary Figure S3.** 14-3-3eta expression in retina. (**a**) Adult (P30) mouse retina immuno-labelled with an anti-14-3-3eta antibody (in green fluorescence) or (**b**) without primary antibody and stained with the nuclear marker Hoechst showing a prominent labeling of RCGs. Scale bars: 50 µm. RGC: retinal ganglion cells, IPL: inner plexiform layer, INL: inner nuclear layer, OPL: outer plexiform layer, ONL: outer nuclear layer.

**Supplementary Figure S4.** Primers used for PCR. (**a**) Mouse primer sequences used for quantitative RT-PCR. (**b**) Human primer sequences used for *YWHAH* screening.
