# Supplementary material for: Loss of function of Ywhah in mice induces deafness and cochlear outer hair cells' degeneration
Source: Cell Death Discov. 2016 Mar 7;2:16017–. doi: 10.1038/cddiscovery.2016.17 (PMC4893315; doi:10.1038/cddiscovery.2016.17)
Supplement: Supplementary Materials and methods [file cddiscovery201617-s6.doc]

**Supplementary Information**

**Materials and Methods**

**14-3-3eta gene trap mice**

Embryonic stem (ES) cells with the “gene trap” insertion in mouse *Ywhah* intron 1 (clone E08H04) were obtained from the German Gene Trap Consortium (GGTC, Neuherberg, Germany). 100 ES cells were injected into C57BL/6J blastocysts and chimera animals were generated and mated to C57BL/6J mice.

**Genotyping**

Genotyping was performed by extracting total DNA from mouse tail with REDExtract-N-AmpTM Tissue PCR Kit (Sigma, Saint-Louis, USA). The following primers have been used: genoYWmF: 5’-TTG-TCA-AGG-TCA-CAC-AGT-GGC-A-3’, genoYWmR: 5’-GCT-GAA-ATC-AGT-ATC-ACC-AAG-G-3’, genoYWLacZ 5’-CAA-GGC-GAT-TAA-GTT-GGG-TAA-GC-3’.

**Mouse electrophysiological exams for vision**

Mice were kept in the animal house facility of the Institute des Neurosciences de Montpellier in clear plastic cages, subjected to standard light cycles (12 h to 90 lux light, 12 h dark). Animals were fed *ad libidum* with a standard rodent diet and treated according to the NIH statement for the use of Animals in Research. Mice were anaesthetized by intra-peritoneal injections of ketamine (120 mg/kg) and xylazine (10 mg/kg). Electroretinograms were performed every two months, using cotton wick recording electrodes [^1^](#_ENREF_1). Visual evoked potentials were recorded using a needle electrode placed subcutaneously near the tail, the reference needle electrode placed subcutaneously in the snout and the recording electrode placed subcutaneously in the back of the head in order to contact the visual cortex of both hemispheres. Three phases of 60 flashes each were performed, then the needle was repositioned and the experiment repeated. The flash duration was 5 ms, with a frequency of 1Hz and intensity of 159 cd.s-1.m-2. The amplitudes obtained during each phase were averaged. The time shift between each phase was approximately 30 seconds. A cut-off filter was set at 35 Hz.

**qRT-PCR**

Each qRT-PCR reaction was performed in triplicate and contained 2.5 μl of RT product (1:5 dilution), 0.5 μM of each primer, 2 μl of 5× LightCycler PCR Mastermix in a final volume of 10 μl and was performed in a Lightcycler 1.5 Instrument (Roche Diagnostics). Quantitative PCR standard curves in 10-fold dilutions and a negative control with PCR-grade water instead of cDNA were included for each target. Relative quantification was performed using L27 as internal reference. After an initial activation step of 10 min at 95°C, 40 cycles consisting of 95°C for 15 s, 60°C for 10 s and 72°C for 15 s were performed. After PCR amplification, a melting curve analysis was generated to check the specificity of the PCR. The relative amounts of cDNA were calculated using the comparative delta-Ct method from two independent experiments.

**PolyPhen-2 prediction**

PolyPhen-2 ([http://genetics.bwh.harvard.edu/pph2/)](http://genetics.bwh.harvard.edu/pph2/) is a tool that predicts the possible impact of an amino acid substitution on the structure and function of a human protein [^2^](#_ENREF_2). The PolyPhen-2 server discriminates nonsynonymous SNPs into three main categories: benign, possibly damaging, or probably damaging (more confident prediction).

**SIFT prediction**

SIFT (Scale Invariant Feature Transform) predicts whether an amino acid substitution affects protein function based on the degree of conservation of amino acid residues in sequence alignments derived from closely related sequences [^3^](#_ENREF_3). The SIFT scores range from 0 to 1, and scores ≤0.05 are predicted by the algorithm to be damaging amino acid substitutions, whereas scores >0.05 are considered to be tolerated. The SIFT tool is at [http://sift.jcvi.org](http://sift.jcvi.org/).

**PROVEAN prediction**

PROVEAN (Protein Variation Effect Analyzer) is a software tool that predicts whether an amino acid substitution has an impact on the biological function of a protein grounded on the alignment-based score [^4^](#_ENREF_4). If the PROVEAN score ≤-2.5, the protein variant is predicted to have a “deleterious” effect, while if the PROVEAN score is >-2.5, the variant is predicted to have a “neutral” effect. The PROVEAN tool is at [http://provean.jcvi.org](http://provean.jcvi.org/).

**Retinal ganglion cell counting**

Cryostat sections of 14 μm were taken in the eye at the point where the optic nerve emerges from the retina from 14-3-3eta^WT/WT^ mice (n=3) and 14-3-3eta^GT/GT^ mice (n=3). The sections were immunolabelled using Hoechst (1/1000, Invitrogen) to visualize nucleus of each cell layers of the retina and Brn3a (1/200, Santa Cruz Biotechnology, Dallas, USA) for specific immunolabelling of retinal ganglion cells (CGRs) and visualized using Zeiss AxioImager microscope. Quantitative data were expressed as the ratio of CGRs/total number of Hoechst immunolabelled cells in the CGR layer.

**References**

1. Chekroud K, Arndt C, Basset D, Hamel CP, Brabet P, Pequignot MO. Simple and efficient: validation of a cotton wick electrode for animal electroretinography. *Ophthalmic Res* 2011, **45**(4)**:** 174-179.

2. Adzhubei IA, Schmidt S, Peshkin L, Ramensky VE, Gerasimova A, Bork P*, et al.* A method and server for predicting damaging missense mutations. *Nature methods* 2010, **7**(4)**:** 248-249.

3. Kumar P, Henikoff S, Ng PC. Predicting the effects of coding non-synonymous variants on protein function using the SIFT algorithm. *Nature protocols* 2009, **4**(7)**:** 1073-1081.

4. Choi Y, Sims GE, Murphy S, Miller JR, Chan AP. Predicting the functional effect of amino acid substitutions and indels. *PloS one* 2012, **7**(10)**:** e46688.
